# LL-37, a Multi-Faceted Amphipathic Peptide Involved in NETosis

**DOI:** 10.3390/cells11152463

**Published:** 2022-08-08

**Authors:** Marko Radic, Sylviane Muller

**Affiliations:** 1 Department of Microbiology, Immunology and Biochemistry, College of Medicine, University of Tennessee Health Science Center, Memphis, TN 38163, USA; 2 CNRS-Strasbourg University Biotechnology and Cell Signaling UMR7242/Strasbourg Drug Discovery and Development Institute (IMS), 67000 Strasbourg, France; 3 University of Strasbourg Institute for Advanced Study (USIAS), 67000 Strasbourg, France

**Keywords:** cathelicidin, cationic antimicrobial protein, NETosis, lupus, neutrophils, structural flexibility

## Abstract

Innate immunity responds to infections and inflammatory stimuli through a carefully choreographed set of interactions between cells, stimuli and their specific receptors. Of particular importance are endogenous peptides, which assume roles as defensins or alarmins, growth factors or wound repair inducers. LL-37, a proteolytic fragment of cathelicidin, fulfills the roles of a defensin by inserting into the membranes of bacterial pathogens, functions as alarmin in stimulating chemotaxis of innate immune cells, and alters the structure and efficacy of various cytokines. Here, we draw attention to the direct effect of LL-37 on neutrophils and the release of extracellular traps (NETs), as NETs have been established as mediators of immune defense against pathogens but also as important contributors to chronic disease and tissue pathogenesis. We propose a specific structural basis for LL-37 function, in part by highlighting the structural flexibility of LL-37 and its ability to adapt to distinct microenvironments and interacting counterparts.

## 1. Introduction

During the last two decades, major intellectual and experimental advances have been made to explore the innate immune system, which, alongside the adaptive immune system, protects vertebrates against external aggressors, and which is dominant in plants, fungi, insects, and primitive multicellular organisms. The innate immune system provides critical mechanisms for the rapid sensing and elimination of pathogens. It is a fast process, a first line of defense, which is characterized by a large but not very specialized spectrum of reactions. In contrast, the adaptive immune system involves antigen-specific responses, which are finely tuned to specific pathogens. Both innate and adaptive immunity include humoral and cellular components that are complex and interaction between them in an interlocking manner.

The innate immune system uses a panoply of effectors; among them are antimicrobial peptides (AMPs) that act as first-barrier host defense molecules. There are over 2000 such peptides that typically contain less than 100 amino acid (aa) residues, and are cationic and amphipathic. Many of the peptides are produced by proteolytic processing from a larger precursor protein. In humans and higher vertebrates, dozens of different proteases distribute into different structural classes and balance with equally diverse protease inhibitors. The result is a continually changing landscape of precursor proteins and proteolytic fragments. There are several classes of AMPs, notably the human cathelicidin family in which hCAP18 is the sole member in humans. This 16 kDa-human cationic antimicrobial protein gives rise to a conserved N-terminal cathelin domain and LL-37, a C-terminal antimicrobial domain that can be released from the precursor protein after cleavage by proteinases (see below).

LL-37 and its murine ortholog mCRAMP (cathelin-related AMP) exhibit a broad spectrum of antimicrobial activity. In addition, LL-37 has attracted increased attention due to its contribution to the development of inflammatory and autoimmune disorders, such as systemic lupus erythematosus (SLE) rheumatoid arthritis (RA), psoriasis, atopic dermatitis and atherosclerosis [1]. LL-37 is expressed in epithelial cells of the testis, skin, the gastrointestinal tract, and the respiratory tract, and in leukocytes such as monocytes, neutrophils, T cells, NK cells, and B cells.

This short editorial aims to briefly report what is known about the structure of LL-37 and its general role in the context of inflammatory and autoimmune diseases. We propose hypotheses rooted in previously published work to put forward several lines of biophysical, biochemical and immunological investigations that may help further decipher the role of this unique peptide. In a more general context, our aim is also to develop some lines of thought on amphipathic proteins and their fragments, which may be central in the pathophysiology and treatment of autoimmune disorders.

## 2. Generation of LL-37 Peptide

**Figure 1:**
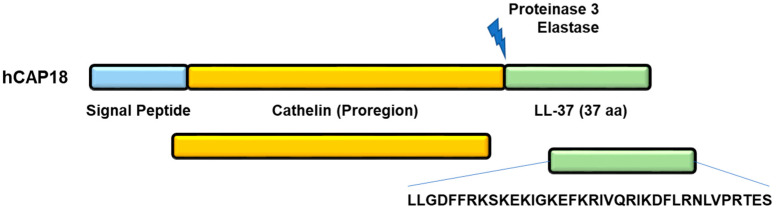
Maturation of hCAP18 precursor protein and naturally occurring generation of the endogenous fragment LL-37.

LL-37 is produced from the hCAP18 precursor protein via proteolytic cleavage by serine proteases kallikreins 5 and 7 (expressed in the epidermis) and proteinase 3 (PR3), mainly expressed in neutrophils. This processing generates the signal peptide (30 aa), the proregion cathelin (103 aa), and the C-terminal domain (37 aa), LL-37, which is active after maturation (Figure 1).

Shorter peptides, such as KR-12 (KRIVQRIKDFLR), for example, that removed aa from LL-37, also share its activity [2]. Some analogues in which certain aa have been replaced, as in KR-12-3 (KRIVKWIKKFLR), for example, or non-cleavable sequences, show enhanced activity compared with LL-37 [3,4].

## 3. Structure of Peptide LL-37

**Figure 2:**
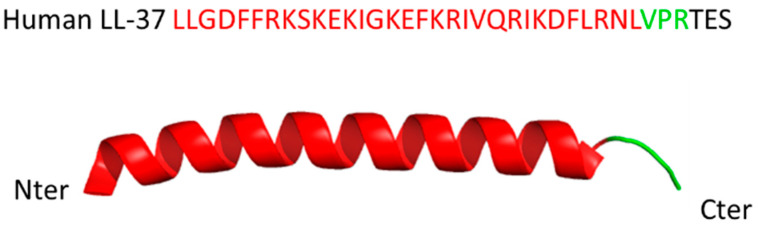
Sequence and ribbon representation structure of human LL-37 (high-quality structure as determined by 3D nuclear magnetic resonance spectroscopy).

LL-37 is a 37 aa-long peptide with two leucine residues at its N-terminus. At neutral pH, it has a total net charge of + 6. It displays amphiphilic properties due to the distribution of 16 charged residues and hydrophilic and lipophilic functional groups in phase with the alpha helix. This unique composition explains the dual affinity of LL-37 for both water and oil (hydrophilic and hydrophobic targets) and its multiplicity of functions. The two- and three-dimensional structure of LL-37 has been resolved in different conditions using full-length and truncated peptide, monomeric and oligomeric forms (dimers, tetramers and fiber-like structures) or peptide complexes with ligands, in different conditions of detergent, lipid and cofactor environment [5,6,7,8,9]. From these studies (crystal and nuclear magnetic resonance), a diversity of structures has emerged highlighting the remarkable flexibility of LL-37 that adapts easily to the interactants it meets [10]. In the presence of a phospholipid bilayer or short-tail phospholipid micelles, for example, LL-37 preferentially adopts an alpha-helical conformation covering residues 2 to 31, with residues 33 to 37 remaining mobile [5] (Figure 2). At micromolar concentrations in water, LL-37 exhibits a circular dichroism spectrum consistent with a disordered structure [11].

Strikingly, the tertiary structure of LL-37 endows the peptide with a wide range of specific and biologically significant interactions that range from potential inhibition of viral infections to intriguing applications in chronic neurodegenerative disorders. Recent examples include the specific and tight interaction between LL-37 and the severe acute respiratory syndrome SARS-CoV-2 Spike protein S1, which, due to the obstruction of S1′s receptor-binding domain, inhibits host cell binding [12]. Demonstrating the versatile nature of LL-37, an alternative binding surface of the peptide interacts with the ligand-binding domain of angiotensin converting enzyme-2 (ACE-2), the receptor for the SARS-CoV-2 viral entry [12]. At the opposite side of the spectrum, LL-37 inhibits the aggregation of α-synuclein, one of the critical contributors to neurodegeneration in Parkinson’s disease (PD) [13]. In this context, the anti-aggregation potency of LL-37 prevents oligomer-induced cellular toxicity and may thus offer a promising avenue to therapy for PD.

## 4. LL-37, Its Central Role in NETosis

One of the first observed properties of LL-37 was its affinity for phospholipid membranes. The binding of LL-37 led to compromised cellular integrity and endowed LL-37 with antimicrobial potency. Thus, it may not be surprising that LL-37 contributes to nuclear membrane disruption and the formation of NETs [14]. This was shown in isolated neutrophils from blood where LL-37 could act alone or along with other known NET stimuli. In addition, LL-37 was observed to transit toward the neutrophil nucleus where it may contribute to the disruption of the nuclear membrane. Upon release of NETs, LL-37 tightly associates with the nuclear chromatin fibers. Due to its positive charge, LL-37 decorates NETs but the association with chromatin reduces the antimicrobial activity of LL-37 [15]. Conversely, NETs coated with LL-37 are resistant to bacterial nuclease degradation and thus may be more active in constraining microbial dispersion.

In vivo testing of the predicted activity of LL-37 in NETosis revealed that LL-37 can be protective in a widely used model for experimental sepsis that involves caecal ligation and puncture (CLP). LL-37 improved the survival of CLP model mice, which may reflect the suppression of macrophage pyroptosis and the reduced release of pro-inflammatory cytokines, such as IL-1β. Importantly, the enhanced release of NETs, possibly aided by LL-37, may have contributed to reduced mortality in the challenged mice by enhancing the bactericidal activity of NETs [16].

The preceding studies suggest a function of LL-37, which follows the release of LL-37 in NETosis and may trans-stimulate NET release in additional neutrophils that arrive at the local site of inflammation. Psoriasis is a skin disease with prominent neutrophil (PMN) infiltration that exhibits high local concentrations of LL37. Plasmacytoid (p) dendritic cells (DCs) are postulated to exacerbate disease following stimulation by LL37 in complex with DNA or RNA. Previously, the initial inflammatory event, considered to involve nucleic acid release in the presence of LL-37, was uncertain. Herster et al., [17] demonstrated that neutrophils mount a self-propagating NET and cytokine release cascade that is independent of DNA but involves RNA. The researchers identified RNA, which is present in NETs and abundant in preparations of psoriatic but not healthy skin, as a partner of LL37. The LL-37/RNA complexes triggered toll-like receptor (TLR)-mediated cytokines and NET release in vitro and in vivo. Addition of LL-37/RNA complexes to naive human PMNs prompted de novo NET release, and thus augmented inflammation. LL-37 in combination with DNA or RNA may contribute to a self-sustaining and amplifying vicious cycle of ongoing and progressive chronic inflammation (see below).

## 5. LL-37 in Lupus

**Figure 3:**
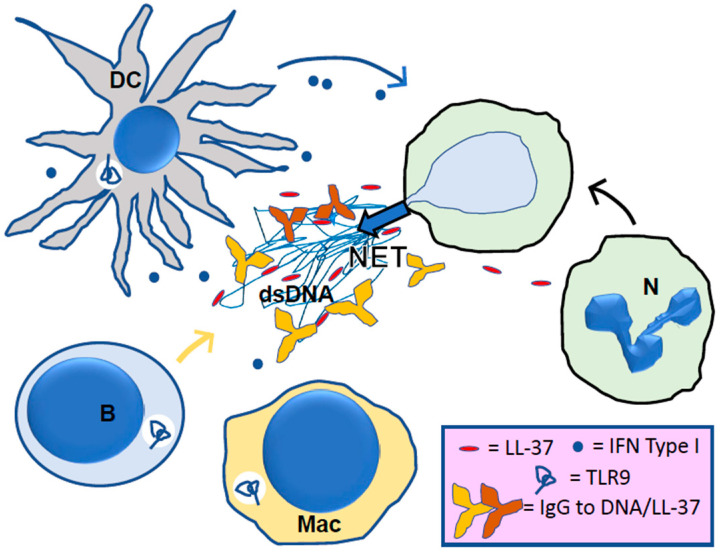
Roles of LL-37 in inflammation and autoimmunity. Neutrophils (N) arrive at site of inflammation and are stimulated to release NETs consisting of double-stranded (ds) DNA associated with histones and numerous other granule components, proteases and myeloperoxidase. The LL-37 peptide is released during NETosis and binds to NET chromatin or diffuses from the site. The LL-37 peptide may serve as chemoattractant for additional neutrophils, macrophage (Mac) or B lymphocytes (B). In autoimmunity, B cells may secrete IgG to dsDNA and to LL-37. Together with NET components, the IgG will be internalized via Fc receptors and stimulate endosomal toll-like receptor 9 (TLR9). In response to TLR9 stimulation, dendritic cells (DC) may secrete type I interferons that further stimulate the immune response.

The effect of LL-37 in SLE has been discussed for more than a decade [1]. In their seminal work, Gilliet and his colleagues explained that in the circulating immune complexes (ICs) that are typically formed in SLE and which are then deposited into peripheral tissues where they trigger detrimental organ inflammation, there are complexes containing extracellular self-DNA and LL-37 peptide derived from NETs [18,19] (Figure 3). These LL-37 peptide-containing complexes, in which LL-37 protects NET-derived DNA from degradation, may contribute to the accumulation of immunogenic NETs and the induction of anti-NET antibodies related to the presence of autoantibodies that, for some, exhibit deleterious properties. Pathogenic autoantibodies against ICs themselves, including Abs to LL-37, occur in patients with SLE [20]. LL-37 antibodies may contribute to ICs (forming LL-37-DNA/anti-DNA complexes) and further raise the production of NETs, leading to the amplification of a vicious inflammatory cycle [19].

LL-37 also assumes roles that are central in innate immunity and autoimmune responses in SLE [20]. As mentioned above, LL-37 peptide-containing complexes are potent stimulators of pDCs, leading to type I interferon production. They can directly activate memory B cells [21]. LL-37 plays other critical roles in lupus via its effects on macrophages and inflammasome [22]. The production of IL1-β and IL-18, boosted by this activation, further amplifies self-propagating inflammation and can therefore be associated with a lupus flare.

As previously stated, RNA interacts with LL-37 in nucleic acid–peptide complexes [17] in which RNA is protected from degradation by RNAses. This finding supported the idea that not only self-, but also foreign (bacterial, viral, fungal)-RNA may associate with LL-37 and exert immunostimulatory effects in this context, a role largely demonstrated and discussed in recent years [23,24], especially in the case of the coronavirus disease 2019 (COVID-19). LL-37 can enhance TLR3 signaling by interacting directly with dsRNA, such as poly (I:C), thus enhancing the type I interferon response [25]. It can form a complex with single-stranded (ss) RNA [26] as it does with ssDNA [18], enhancing TLR7/TLR8 and TLR9 signaling, respectively, that play pivotal roles in SLE [27,28,29].

Although LL-37 exerts a functional role in SLE pathophysiology, it remains a matter of debate as to whether serum LL-37 represents a biomarker of lupus disease activity. Conflicting data have been described in the literature showing an association or absence of correlation with lupus manifestations [30,31,32]. Many factors could explain these discrepancies and, among them, the type of assay applied to quantify the levels of LL-37. Using screening tests that discriminate the different forms of LL-37, which is subjected to irreversible post-translational modifications, such as citrullination and carbamylation [33], may be valuable in this respect [34]. Further studies are thus warranted to determine if LL-37 could be a biomarker of interest in SLE.

## 6. LL-37 in other Autoimmune and Inflammatory Settings

Recent examples in the literature have revealed the effects of LL-37 in many other inflammatory settings. The effects comprise the ability of LL-37 to neutralize lipopolysaccharide, alter membrane integrity, stimulate migration of cells of the innate immune system, affect DC maturation, stimulate production of cytokines and chemokines, interact with their receptors, and trigger mast cell degranulation. It is not surprising, therefore, that LL-37 acts as a trigger and a major perpetuation factor in a large range of autoimmune/inflammatory diseases. Its impacts in psoriasis, RA, Felty’s syndrome, polymyositis and dermatomyositis, type I diabetes, atherosclerosis, neurodegenerative diseases such as PD, and other settings, have been explored and documented [23,35,36,37]. Although some data remain difficult to reconcile or are enigmatic due to inconsistent or incomplete cellular, molecular and genetic observations, other studies describe more mechanistic results that help to understand the mode of action of LL-37 in these different settings. Thus, Minns et al. [38] described data in psoriasis that support the recognition of LL-37 as an antigen by CD4^+^ T cells, which in turn produce more IL-17, a critical cytokine in this disease. In this study, LL-37 was shown to be a potent Th17 cell activator, which protects Th17 but not Th1 cells from apoptosis. In RA, elevated expression of LL-37 was demonstrated. LL-37 induces the apoptosis of osteoblasts, which amplifies the defect in bone formation in arthritic joints [39]. Intriguingly, in type I diabetes, Parackova et al. showed that, compared to healthy donors, patients’ NETs contained significantly more DNA–histone complexes and neutrophil elastase but less myeloperoxidase (known to be positively associated with obesity and diet-induced insulin resistance) and LL-37 [40]. It is not known how this molecular variation affects clinical parameters.

LL-37 has also caught the attention in the field of altered fibrosis [36]. Particularly in a pulmonary disease context, it has been demonstrated that LL-37 is highly expressed in small airway epithelium from patients with chronic obstructive pulmonary disease (COPD). The magnitude of LL-37 expression in epithelium was positively correlated with airway wall thickness and collagen deposition [41]. Co-localization of LL-37 and collagen in these tissues correlated with findings obtained with the fibroblast cell line HFL-1, suggesting that LL-37 promotes fibroblast collagen production through formyl peptide receptor-like 1 (FPRL1)-dependent extracellular signal-regulated kinase signaling pathway. This mechanism may contribute to small airway remodeling in COPD [42]. It should be mentioned that LL-37, but not the murine ortholog mCRAMP, stimulates signaling by poly (I:C) through a FPRL1-dependent pathway [43], a feature that may result from sequence and structural differences in the two peptides.

A decreased production of LL-37 from hCAP-18 in neutrophils has important consequences. A rare autosomal recessive disease caused by mutations in the gene encoding lysosomal cathepsin C, for example, was found in patients with Papillon-Lefèvre syndrome (PLS) [44]. Proteome profiling of isolated cellular fractions showed that neutrophils of these patients lack (or exhibit reduced levels of) several types of proteases, including PR3. Lack of PR3 and deficient processing of hCAP-18 in neutrophils may play a role in the propensity of patients with PLS to develop periodontal disease [45]. It is known that neutrophils and NETs play a crucial role in preventing periodontitis [46]. Analyzing in depth the role of LL-37 in this context could generate important information that may help develop new effective treatments for this and other infectious diseases.

## 7. Final Comments and Driving Forces

Nowadays, the role of LL-37 in chronic (auto)inflammatory diseases still remains a matter of debate due to contradictory data and missing mechanistic information. Factors that complicate in depth investigations include conditions of how LL-37 effects are measured, the type of peptide that was used (knowing that this natural peptide is subjected to post-translational modifications that change its functional properties), the medium in which the activity of LL-37 is evaluated, the possible presence of anti-LL-37 antibodies in the fluid of interest and other experimental details.

The LL-37 structural flexibility is a decisive property of this natural peptide. Depending on the ligand to which LL-37 associates, its structure changes and it cannot be followed upon the same criteria of detection. LL-37 effectively belongs to a very large family of proteins better known as intrinsically disordered proteins/peptides (IDPs), the native state is best characterized as a dynamic ensemble of interconverting conformations. IDPs are often composed of modules with flexible bridges between the modules. IDPs show pleiotropic functions upon the partner they bind to. This is the case of, among many others, alpha-synuclein, Tat (trans-activator of transcription) protein, Tau protein, tumor suppressor p53, and the chaperone HSPA8 protein [47,48]. In the past, little attention was given to this class of proteins and peptides with the argument that unstructured interactions between proteins and other molecules or surfaces are nonspecific, and therefore have no decisive role in biology. In fact, these proteins and natural peptides, once bound to a particular ligand or receptor, show interactions that are very selective and of high affinity or avidity.

A final question to be raised to conclude this short review is whether LL-37 could constitute an active principle for therapy. Nowadays, more and more peptides have been accepted by the agencies for therapeutic applications in diverse pathological areas [49]. However, LL-37 is an endogenous peptide that is produced naturally. This consideration complicates its protection in terms of patent and commercial exploitation. Beyond this, some safety considerations were raised. For example, neutralizing LL-37 with induced antibodies to LL-37 could be advantageous in autoimmune and chronic inflammatory diseases but could also compromise antimicrobial defenses [50]. At this stage of our knowledge, the therapeutic benefits of LL-37 warrant much further investigation. A great advantage regarding LL-37 is that this peptide (and analogues) can be easily produced by solid-phase synthesis, which guarantees its production in large quantity and controlled fashion, following good manufacturing practice (GMP) requirements. Future research will indicate the interest we may have to up- or down regulate its natural production, administer exogenous peptide or on the contrary, to diminish its levels in the circulation or in targeted tissues. As for any immunosuppressive and immunomodulatory therapeutic, it will be decisive to evaluate the long-term consequences of LL-37 on infection, vascularization/angiogenesis, cancer and autoimmunity.
